# Super enhancer inhibitors suppress MYC driven transcriptional amplification and tumor progression in osteosarcoma

**DOI:** 10.1038/s41413-018-0009-8

**Published:** 2018-04-04

**Authors:** Demeng Chen, Zhiqiang Zhao, Zixin Huang, Du-Chu Chen, Xin-Xing Zhu, Yi-Ze Wang, Ya-Wei Yan, Shaojun Tang, Subha Madhavan, Weiyi Ni, Zhan-peng Huang, Wen Li, Weidong Ji, Huangxuan Shen, Shuibin Lin, Yi-Zhou Jiang

**Affiliations:** 1 The Department of Dermatology, Yale University, New Haven, CT 06510 USA; 2 Department of Musculoskeletal Oncology, The First Affiliated Hospital, Sun Yat-sen University, Guangzhou, 510080 Guangdong China; 3 State Key Laboratory of Ophthalmology, Zhongshan Ophthalmic Center, Sun Yat-sen University, Guangzhou, 510060 Guangdong China; 4 Institute for Advanced Study, Shenzhen University, Shenzhen, 518060 Guangdong China; 5 Innovation Center for Biomedical Informatics, Georgetown University Medical Center, Washington, DC 20007 USA; 6 Department of Oncology, Georgetown University Medical Center, Washington, DC 20007 USA; 7 Department of Pharmaceutical and Health Economics, School of Pharmacy, University of Southern California, Los Angeles, CA 90089 USA; 8 Center for Translational Medicine, The First Affiliated Hospital, Sun Yat-sen University, Guangzhou, 510080 Guangdong China; 9 Department of Surgery, The First Affiliated Hospital, Sun Yat-sen University, Guangzhou, 510080 Guangdong China; 10 Biobank of Eye, State Key Laboratory of Ophthalmology, Zhongshan Ophthalmic Center, Sun Yat-sen University, Guangzhou, 510060 Guangdong China

## Abstract

Osteosarcoma is the most common primary bone sarcoma that mostly occurs in young adults. The causes of osteosarcoma are heterogeneous and still not fully understood. Identification of novel, important oncogenic factors in osteosarcoma and development of better, effective therapeutic approaches are in urgent need for better treatment of osteosarcoma patients. In this study, we uncovered that the oncogene MYC is significantly upregulated in metastastic osteosarcoma samples. In addition, high MYC expression is associated with poor survival of osteosarcoma patients. Analysis of MYC targets in osteosarcoma revealed that most of the osteosarcoma super enhancer genes are bound by MYC. Treatment of osteosarcoma cells with super enhancer inhibitors THZ1 and JQ1 effectively suppresses the proliferation, migration, and invasion of osteosarcoma cells. Mechanistically, THZ1 treatment suppresses a large group of super enhancer containing MYC target genes including CDK6 and TGFB2. These findings revealed that the MYC-driven super enhancer signaling is crucial for the osteosarcoma tumorigenesis and targeting the MYC/super enhancer axis represents as a promising therapeutic strategy for treatment of osteosarcoma patients.

## Bone cancer: Disease-promoting DNA regions revealed as therapeutic targets

Insight into a molecular pathway involved in an aggressive bone cancer has suggested a new approach to its treatment. Osteosarcoma usually develops in growing bone tissue, but often spreads to other organs, consequently reducing survival. The molecular mechanisms behind osteosarcoma are not fully understood. A team led by Shuibin Lin from Sun Yat-sen University investigated the role of *MYC*, a gene that is important in other cancers. They found that increased expression of *MYC* is associated with the spread of osteosarcoma and a poor prognosis because the protein product of *MYC* activates many super-enhancers, regions of DNA that increase the expression of cancer-related genes. Inhibition of the super-enhancers suppressed growth and spreading of osteosarcoma in cultured cells and a mouse model, suggesting a novel therapeutic approach to osteosarcoma.

## Introduction

Osteosarcoma is the most common type of primary bone sarcoma that mostly occurs in teenagers and young adults.^1^ Osteosarcoma usually grows in the fast-growing bone tissues but often metastasizes into other organs such as lung. It’s a highly aggressive tumor that causes intense pain, decreased range of motion, swelling, and fragile bones in patients. The overall 5 year survival rate for the non-metastatic disease is about 60%–70%, while the survival rate dramatically decreases in patients with metastases and recurrent diseases.^2,3^ The causes of osteosarcoma are heterogeneous and still not fully understood, therefore a better understanding of the molecular mechanisms underlying osteosarcoma oncogenesis and identification of novel therapeutic targets are urgently needed for the effective treatment of osteosarcoma.

Aberrant growth hormones levels, genetic and epigenetic misregulations are important osteosarcoma-associated risk factors.^2,3^ Genetic mutations of tumor suppressor genes TP53, RB1, and DNA helicase genes (RECQL4, RECQL4, WRN, and BLM) are frequently found in osteosarcoma patients.^2,3^ In addition, misregulated expressions of oncogenes such as MYC and c-fos are often associated with osteosarcoma oncogenesis and progression.^4^ MYC proto-oncogene promotes oncogenic transcriptional amplification program in cancers and represents as an important therapeutic target for cancer therapy.^5,6^ MYC gene has been reported to be amplified in osteosarcoma and its expression is often upregulated in osteosarcoma patients.^4,7^ MYC overexpression together with the loss of Ink4a/Arf can promote the transformation of bone marrow stromal cells into osteosarcoma.^8^ Most importantly, high-level of MYC is associated with low apoptosis and poor prognosis of osteosarcoma patients.^4,8,9^

MYC mediated transcriptional amplification through super enhancers is an important hallmark of cancer.^10^ Super enhancers are a group of strong enhancer regions that each contains clusters of enhancer elements bound by multiple transcription factors to facilitate the strong expression of cell identify related genes and play important function in cancers and diverse biological processes such as retinal pigment epithelium plasticity.^11–13^ Super enhancers are characterized by strong mediator protein binding and H3K27 acetylation (H3K27ac).^11,12^ Small molecules such as cyclin-dependent kinase 7 (CDK7) inhibitor THZ1 and bromodomain Inhibitor JQ1 preferentially inhibit super enhancer signaling and have been widely used to treat diseases with misregulated super enhancer activities.

In this study, we investigated the molecular mechanisms underlying MYC amplification and overexpression in regulation of osteosarcoma. We found that MYC preferentially regulates the super-enhancer containing genes and mediates the transcriptional amplification of its target genes in osteosarcoma. We further revealed that THZ1 and JQ1 can efficiently suppress the MYC driven transcriptional amplification and inhibit the osteosarcoma growth, migration, and invasion. Our data uncovered novel mechanisms of MYC oncogene in regulation of osteosarcoma and provided important insights for the diagnostics and therapy of osteosarcoma patients.

## Results

### MYC expression is associated with poor prognosis of osteosarcoma

**Fig. 1:**
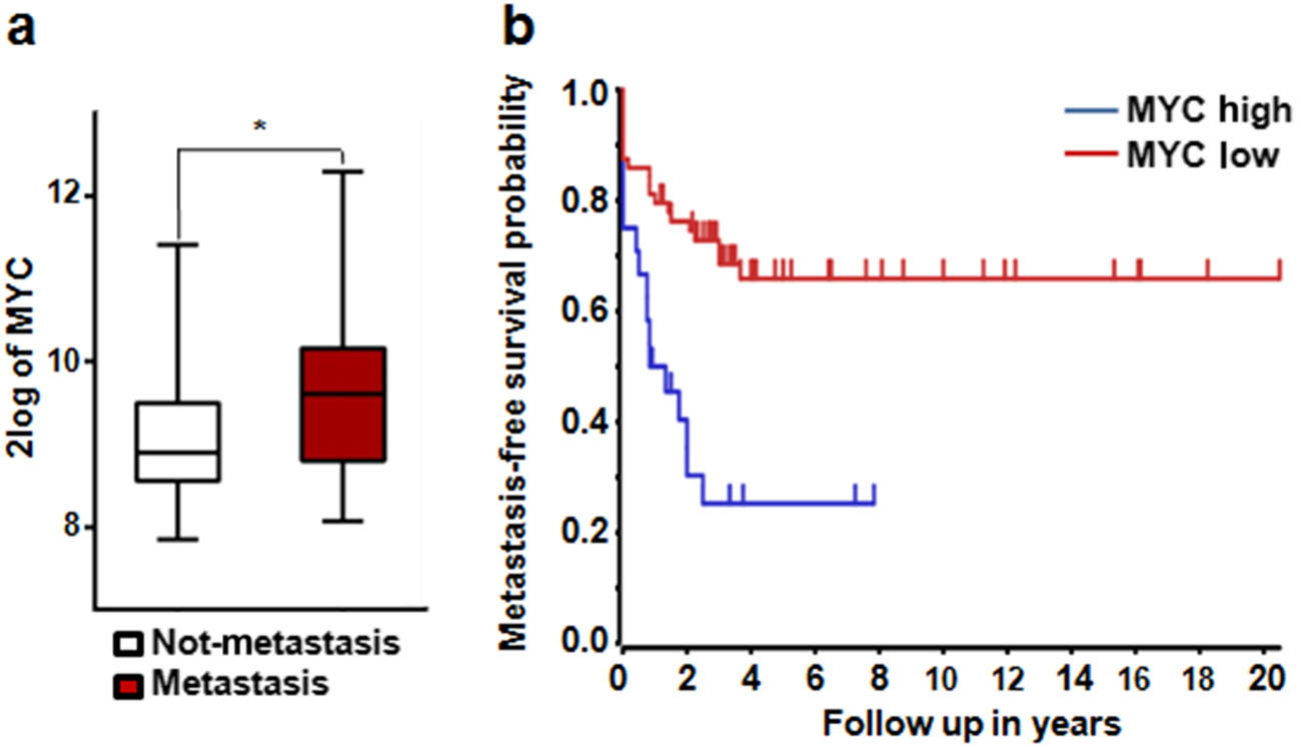
MYC expression is associated with poor prognosis of osteosarcoma. **a** Comparison of MYC expression in metastasis and non-metastasis osteosarcoma patient samples. MYC expression levels in patient samples were analyzed using the mixed osteosarcoma—Kuijjer dataset at R2: Genomics Analysis and Visualization Platform (http://r2.amc.nl).^14^ *P < 0.05. **b** Association between MYC expression and osteosarcoma patient survival. *P* = 2.6 e^−4^.

To study potential role of MYC in regulation of osteosarcoma, we first determined the association between MYC expression level and osteosarcoma patient prognosis using a published osteosarcoma patient transcriptome profiling dataset.^14^ We found that the expression of MYC is significantly up-regulated in the metastatic osteosarcoma patient samples compared to those non-metastasis samples, suggesting that MYC expression might promote the metastasis of osteosarcoma (Fig. 1a). In addition, patient survival analysis revealed that the patients with high MYC expression have dramatically shorter survival time than patients with low MYC expression, supporting the negative association between MYC expression and patient survival (Fig. 1b). Overall, these data revealed that MYC expression is associated with progression and poor prognosis of osteosarcoma.

### MYC regulates super enhancer containing genes in osteosarcoma

**Fig. 2:**
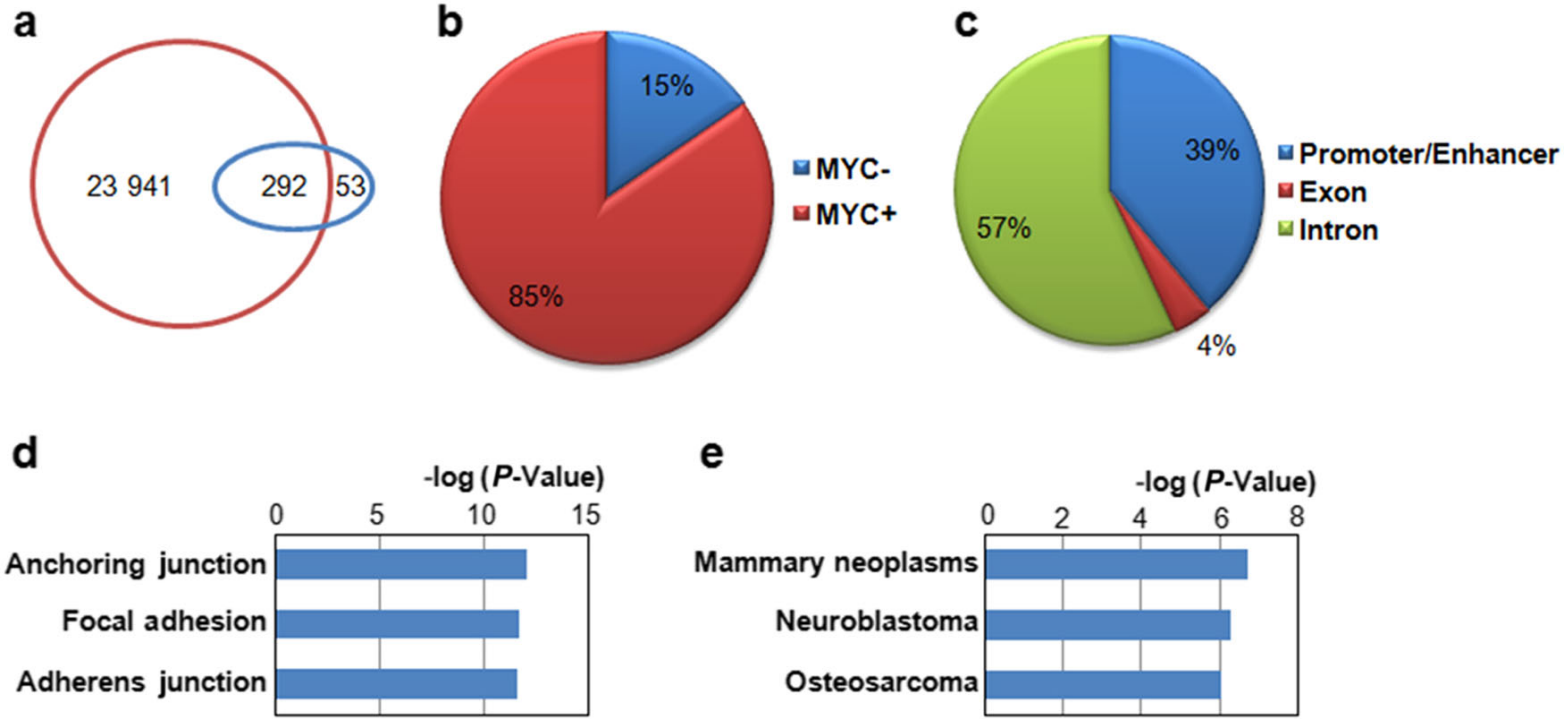
MYC regulates super enhancer genes in osteosarcoma. **a** Overlap between MYC binding sites and super enhancer regions in U2OS cells. Brown circle represents the size of MYC target; blue circle represents the size of super enhancers. **b** Groups of super enhancer regions based on MYC binding status. The super enhancers were separated into two different groups based on their MYC binding statuses. **c** Distribution of MYC bound super enhancers in different genomic regions. **d**, **e** Gene ontology analysis of MYC bound super enhancer associated genes. **d** Cellular component gene ontology; **e** disease gene ontology (color figure online).

Next we studied the potential molecular mechanisms underlying MYC’s function in osteosarcoma. First, we analyzed the MYC target genes in osteosarcoma using the published ChIP-Seq data.^15^ Analysis of MYC ChIP-Seq data in U2OS cells identified more than 24 000 MYC binding sites (Fig. 2a), suggesting that MYC regulates a large number of targets in the osteosarcoma cells. Since it has been reported that MYC amplifies transcription by preferentially targeting super enhancer genes, we compared the MYC target sites with the super enhancer regions identified by H3K27ac ChIP-Seq in the U2OS cells^16^ and found that MYC binds to about 85% of the super enhancer regions (Fig. 2a, b), confirming the role of MYC in regulation of super enhancer genes. In addition, those MYC bound super enhancer regions are mainly located in the enhancer/promoter and the intron regions (Fig. 2c). Interestingly, gene ontology analysis revealed that MYC regulated super enhancer genes are the signature genes that function in regulation of anchoring junction/adhesion (Fig. 2d), which includes genes that are essential during the process of epithelial–mesenchymal transition (EMT) and tumor metastasis. Moreover, disease gene ontology analysis revealed that the MYC regulated genes are the signature genes in mammary neoplasms including osteosarcoma (Fig. 2e), suggesting the potential role of MYC in regulation of cancer cell adhesion and invasion.

### MYC inhibition using super enhancer inhibitors suppresses osteosarcoma proliferation and increases cellular apoptosis

**Fig. 3:**
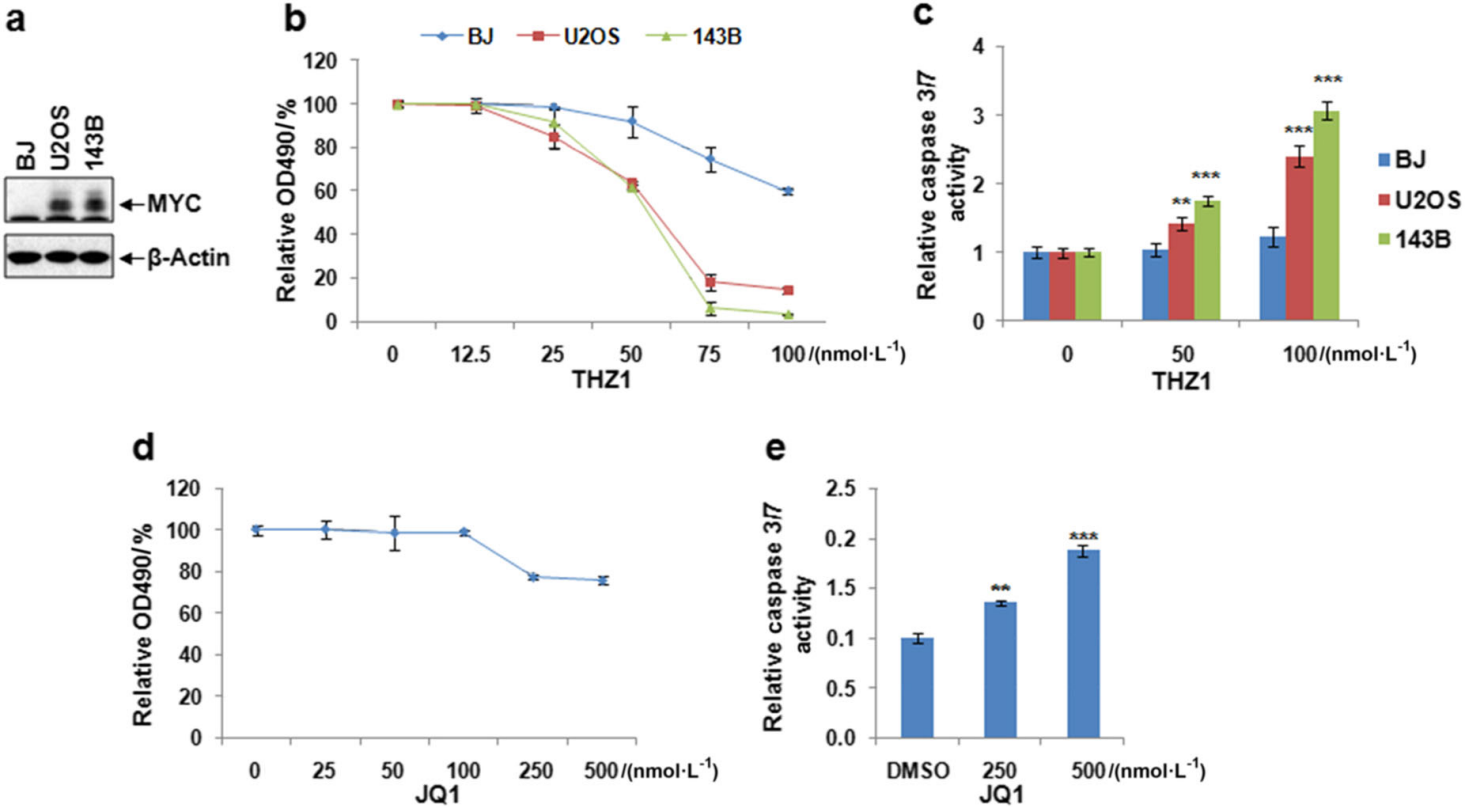
Super enhancer inhibitors suppress osteosarcoma proliferation and induce apoptosis. **a** Western blotting of MYC expression in different cells, β-Actin was used as loading control. **b** MTS assay of U2OS, 143B, and BJ cell proliferation under treatment of THZ1. Cells were treated with different concentration of THZ1 and DMSO and planted into 96-well cell culture plate at 1 000 cells/well, then cell proliferation was measured by OD490 at day4 after treatment. **c** Cells were treated with indicated concentration of THZ1 or DMSO for 48 h, then the cell apoptosis was measured by the Caspase 3/7 activities using the Caspase-Glo® 3/7 Assay Systems. **d** MTS assay of U2OS proliferation under treatment of JQ1. **e** analysis of apoptosis in U2OS cells treated with JQ1. *n* = 3. **P < 0.01, ***P < 0.001.

The CDK7 inhibitor THZ1 and BRD4 inhibitor JQ1 can efficiently target MYC driven transcriptional amplification in other types of cancers, here we studied the potential role of THZ1 and JQ1 in regulation of ostesosarcoma. We first compared the expression of MYC in two osteosarcoma cell lines (U2OS and 143B) and a control human fibroblast cell line (BJ), our result uncovered that MYC expression is up-regulated in osteosarcoma cells compared to the control fibroblasts (Fig. 3a). Treatment of the osteosarcoma and BJ cells with different concentrations of THZ1 revealed that THZ1 can efficiently inhibit the proliferation and increase apoptosis in osteosarcoma cells, while the BJ fibroblasts cells are relatively insensitive to THZ1 treatment (Fig. 3b, c). These data suggested that THZ1 preferentially causes apoptosis in osteosarcoma cells and therefore could be used to target osteosarcoma with minimal effect on the normal cells. THZ1 treatment shows better inhibitory effect compared to the JQ1 treatment (Fig. 3d, e), suggesting that THZ1 is a better inhibitor for the suppression of osteosarcoma proliferation. These data suggested that THZ1 preferentially causes apoptosis in osteosarcoma cells and therefore could be used to target osteosarcoma with minimal effect on the normal cells.

### Super enhancer inhibitors delay osteosarcoma cell migration and invasion

**Fig. 4:**
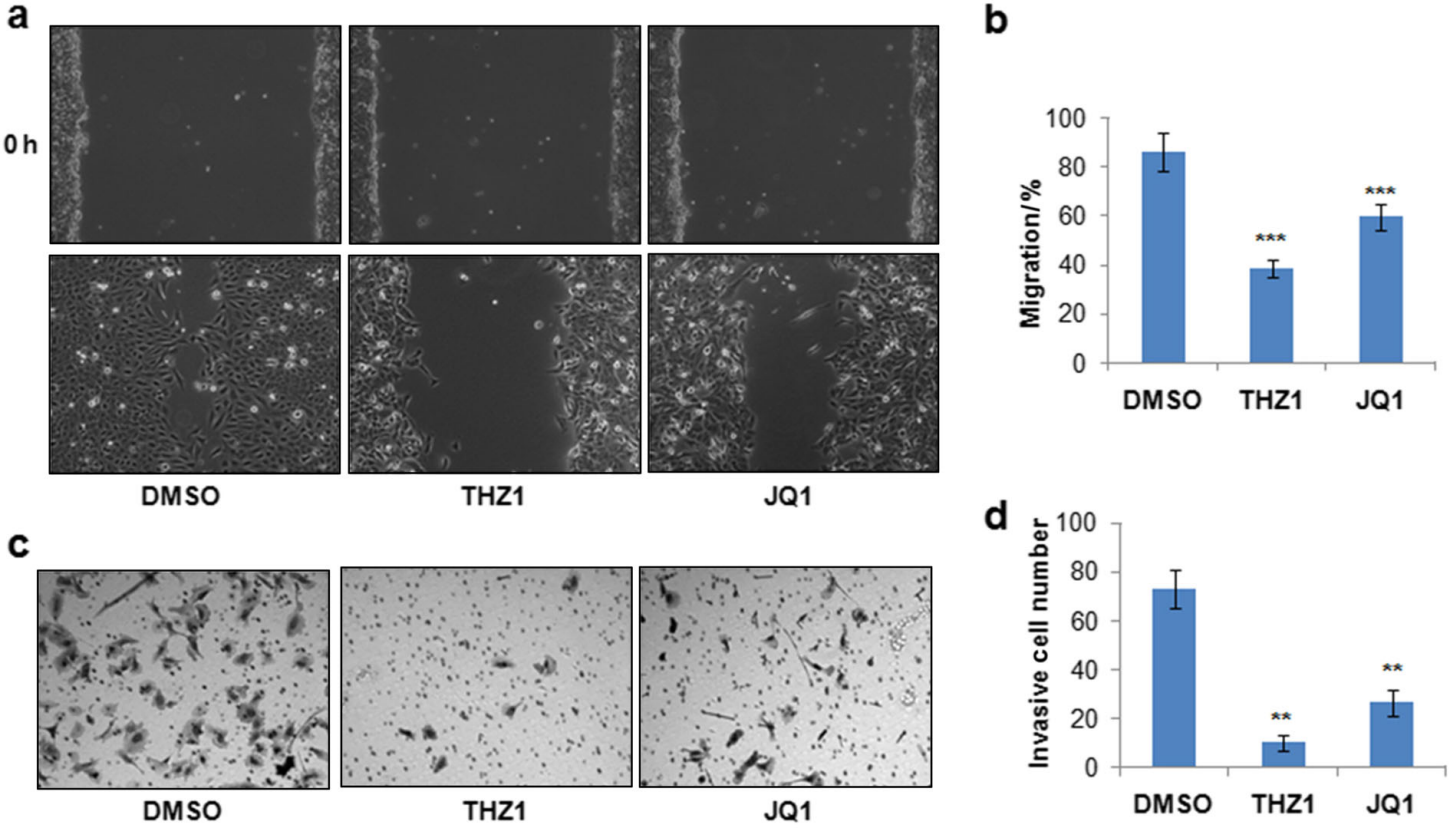
Super enhancer inhibitors suppress osteosarcoma migration and invasion. **a** U2OS cells were cultured in regular mediumto 100% confluence and then further cultured another 24 h in low-serum (2%) medium. Then scratches were induced and cells were treated with 50 nmol·L^-1^ THZ1 and 250 nmol·L^-1^ JQ1 in low-serum medium.Cell images were then acquired to monitor the cell migration after 24 h. **b** Quantification of cell migration (**a**). **c** U2OS osteosarcoma cells were treated with 500 nmol·L^-1^ JQ1, 100 nmol·L^-1^ THZ1 or DMSO for 24 h, then 50 000 cells were seeded into the Matrigel Invasion Chamber. The cell images and invasive cells were acquired and quantified after 16 h. **d** Quantification of invasive cells (**c**). *n* = 3, **P < 0.01, ***P < 0.001.

Given that MYC regulating super enhancer genes function in cellular junction and adhesion, we further studied the role of super enhancer inhibitors in regulation of U2OS osteosarcoma cell migration and invasion. U2OS cells were first treated with THZ1 or JQ1 for 24 h and then subjected to wound healing assay to study the roles of THZ1 and JQ1 in scratch induced cell migration. Our result showed that both THZ1 and JQ1 treatment delays the wound healing in U2OS cells, and THZ1 showed stronger effect than JQ1 (Fig. 4a, b), suggesting that super enhancer inhibitors significantly suppress the migration capacity of U2OS osteosarcoma cells. We further determined the role of THZ1 and JQ1 in the invasion of U2OS cells, as presented in Fig. 4c, d, THZ1 and JQ1 treatment significantly inhibits the invasion of U2OS cells, and the inhibitory effect is better than the JQ1 treatment. Overall, the above data uncovered that THZ1 and JQ1 are effective compounds that can strongly inhibit the proliferation, migration and invasion of osteosarcoma cells.

### THZ1 inhibits osteosarcoma growth in vivo in xenograft tumor model

**Fig. 5:**
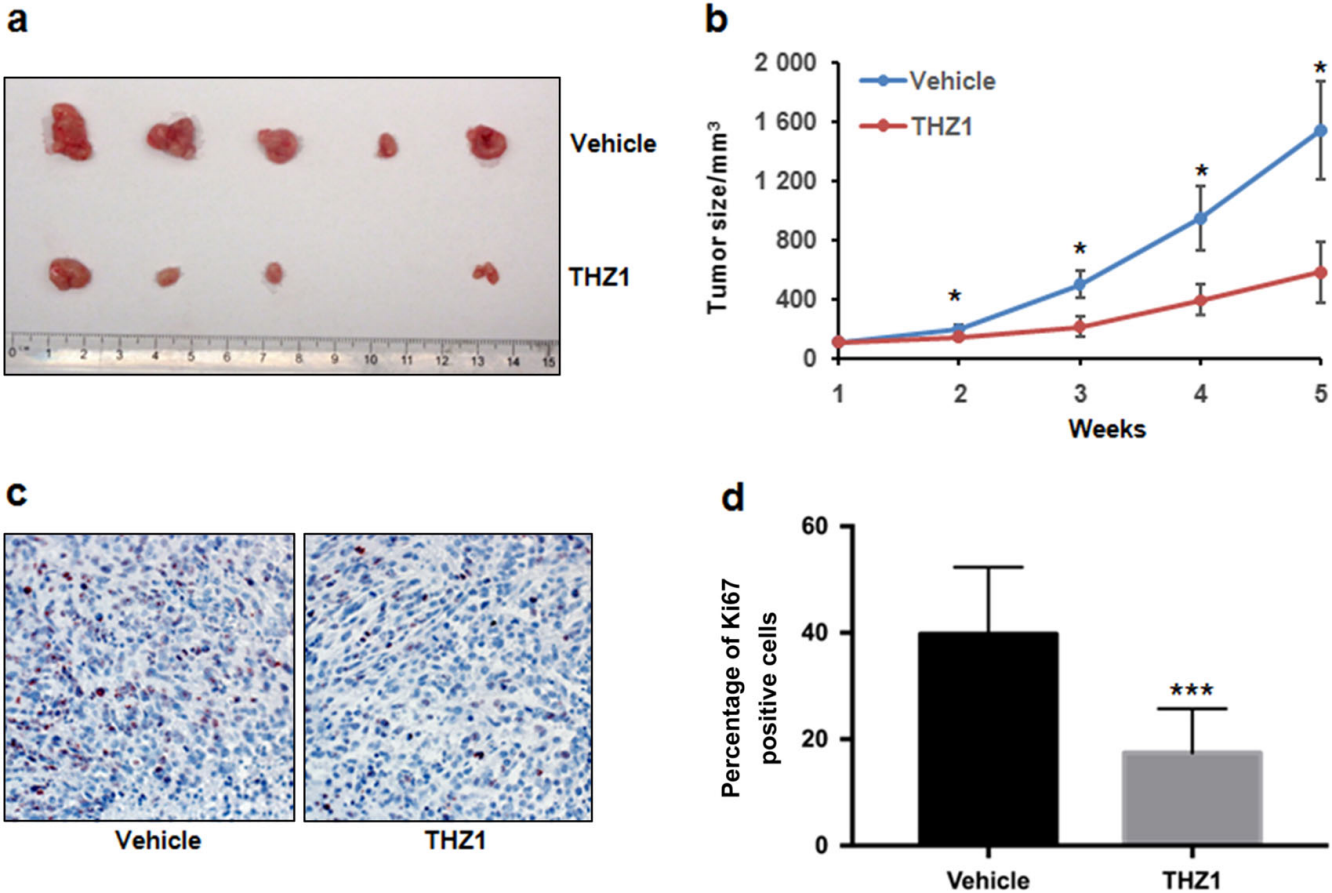
THZ1 inhibits 143B tumor growth in xenograft mice model. **a**, **b** Tumor growth of 143B xenografts (5 mice/group) treated with vehicle control or THZ1 were measured to evaluate the effect of THZ1. Data represent mean tumor volume ± SEM of different treatment groups (blue: vehicle control; brown: THZ1). Statistical significance was determined by Student *t*-test. *P < 0.05. **c** Immunohistochemistry staining of Ki67 in xenografts from subcutaneous transplantation of 143B cells into mice administered with vehicle control or THZ1. **d** The proportions of cells with positive staining of Ki67 in xenografts werequantified. Statistical significance was determined by Student *t*-test. ***P < 0.001.

To assess the in vivo anti-osteosarcoma ability of THZ1, we used a nude mouse xenograft model with143B cells. Control vehicle or THZ1 were subcutaneously injected into nude mice twice a day. Our results showed that THZ1 administration significantly suppressed tumor growth when compared with control (Fig. 5a). At the end point of our study, the average volumes of the tumors were 1 544 mm^3^ for the control and 585 mm^3^ for the THZ1 treatment group (Fig. 5b). In addition, the expression of the cell proliferation marker, Ki67, was also dramatically decreased in THZ1-treated tumor cells (Fig. 5c, d). These results demonstrated that THZ1 possesses antitumor properties and can suppress tumor proliferation in human osteosarcoma cells in vivo.

### Super enhancer inhibitor THZ1 suppresses MYC target genes in osteosarcomacells

**Fig. 6:**
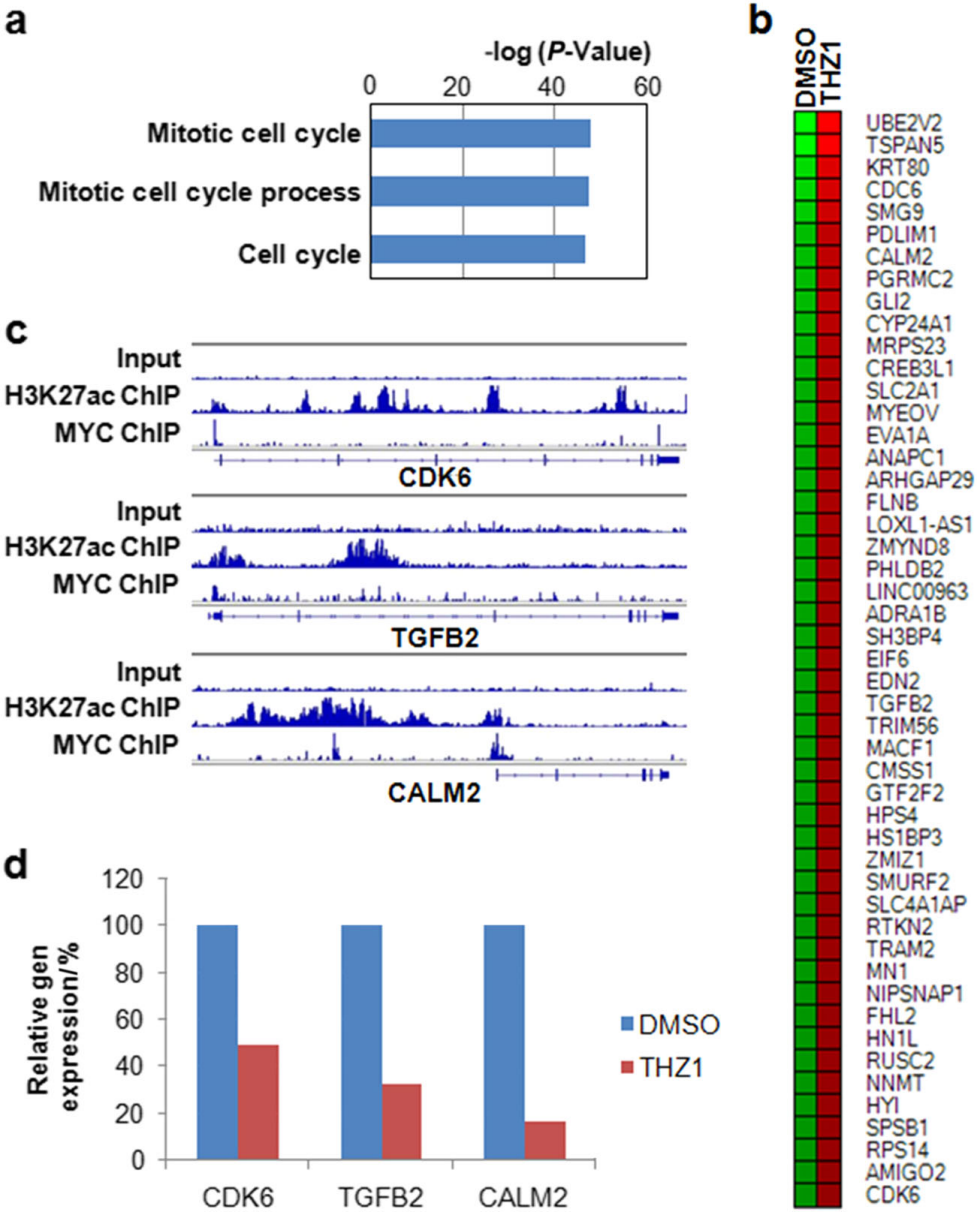
THZ1 suppresses MYC regulated super enhancer genes. **a** Gene ontology of the downregulated genes after THZ1 treatment. **b** Heat map of MYC bound super enhancer genes. **c** Integrative genomics viewer (IGV) plots of representative genes. The blue boxes represent exons, and blue lines represent introns. **d** Relative expression of selected genes (color figure online).

We further studied the molecular mechanisms of THZ1 in regulation of osteosarcoma. To do this, we performed global profiling of gene expression using the RNA samples isolated from U2OS cells that were treated with THZ1 or dimethyl sulfoxide (DMSO) control. Our RNA sequencing result revealed that THZ1 treatment decreases the expression of large number of genes, with 2802 genes expression decreased for more than two-fold. Gene ontology revealed that the downregulated genes function in cell cycle progression (Fig. 6a), which is consistent with our finding that THZ1 treatment resulted in the decreased proliferation of osteosarcoma cells (Fig. 3a). Moreover, many MYC bound super enhancer genesincluding CDK6, TGFB and CALM2 are downregulated upon THZ1 treatment (Fig. 6b–d), further confirming that role of THZ1 in targeting super enhancer signaling in osteosarcoma.

## Discussion

Despite the recent progress in osteosarcoma research, the causes of osteosarcoma are still not fully understood and the development of novel therapeutic strategies for osteosarcoma is in urgent need. In this study, we revealed that MYC expression is significantly elevated in metastatic osteosarcoma samples, and most importantly, high MYC expression is associated with poor patient survival, suggesting that MYC could be an important therapeutic target for osteosarcoma treatment. The MYC regulated super enhancer genes are the mammary neoplasms signature genes function in anchoring junction/adhesion, which is consistent with MYC’s function in regulation of cancer invasion. We further tested the recently developed small molecule super enhancer inhibitors in osteosarcoma and found that super enhancer inhibitors effectively inhibit the proliferation, migration and invasion of osteosarcoma. Mechanistically, THZ1 treatment suppresses a large group of super enhancer containing MYC target genes. Interestingly, not all the MYC bound super enhancers are equally regulated by THZ1 treatment (Supplementary Table 1), some super enhancer genes are more sensitive to THZ1 treatment than others, suggesting that factors such as the MYC binding intensity, super enhancer size and intensity, the basal gene expression levels, differential transcription factor and co-factors activities might account for the differential responses of super enhancer genes to the THZ1 treatment. Overall, our data suggested that super enhancer inhibitors are promising small molecules that can target the MYC signaling in osteosarcoma and therefore could be used as therapeutic drug for the treatment of osteosarcoma patients.

In many different types of cancers, the MYC and MYCN preferentially drive the strong expression of super enhancer containing oncogenes to drive cancer progression, and it has been reported that targeting MYC driven super enhancer signaling is a promising and effective strategy for cancer therapy.^3,17,18^ Here in this study, we studied the potential role of MYC in osteosarcoma and found that MYC is aberrantly expressed in osteosarcoma patients and MYC expression is associated with poor osteosarcoma prognosis, therefore, our data identified MYC as a potential therapeutic target for osteosarcoma.

Super enhancer inhibitors such as JQ1 and THZ1 can selectively and effectively target the MYC driven transcriptional amplification in cancers.^3,17–19^ Different from the BRD4 inhibitor JQ1, THZ1 is a covalent CDK7 inhibitor that can efficiently suppress MYC mediated transcriptional amplification of super enhancer genes at lower concentration.^3^ THZ1 treatment can successfully inhibit the in vitro and in vivo growth multiple types of cancers including the eye cancer retinoblastoma,^24–26^ high-grade glioma,^20^ breast cancer,^21^ ovarian cancer,^22^ T-cell lymphomas,^23^ esophageal squamous cell carcinoma,^24^ small cell lung cancer,^25^ and neuroblastoma.^18^ Here, we determined the role of THZ1 in osteosarcoma and found that THZ1 can suppress the proliferation, migration and invasion of osteosarcoma cells through inhibiting the expression of important super enhancer genes. Of the THZ1 downregulated target super enhancer containing genes, many of them are critical oncogenes for osteosarcoma, for example, it has been reported that TGFB and CDK6 are important regulators of osteosarcoma proliferation and migration.^26,27^ Therefore, THZ1 inhibits osteosarcoma proliferation, migration, and invasion by targeting super enhancer containing oncogenic genes.

Overall, our results uncovered the important role of MYC in regulation of osteosarcoma metastasis and patient survival. The targeting MYC driven super enhancer signaling using THZ1 represents as a promising therapeutic strategy for the treatment of osteosarcoma.

## Materials and methods

### Cell culture

The osteosarcoma cell lines U2OS, 143B and the human fibroblast cell line BJ was cultured using DMEM medium plus 10% fetal bovine serum (FBS) and antibiotics (100 μg·mL^-1^ Penicillin-Streptomycin). Cells were cultured in the incubator at 37°Cin a humidified atmosphere of 5% CO_2_ and 95% air.

### Analysis of MYC expression in patient osteosarcoma samples

The expression of MYC in osteosarcoma patient samples was derived from the R2: Genomics Analysis and Visualization Platform (http://r2.amc.nl) using the Mixed Osteosarcoma—Kuijjer dataset.^14^ The expression levels of MYC in individual patient samples were downloaded and then analyzed using Prism 6 to compare the MYC expression between the metastasis and non-metastasis osteosarcoma samples. To determine the association between MYC expression and patient survival status, Kaplan Meier survival curve was generated using the same dataset with default setting in R2 Platform, which uses every increasing expression value as a cutoff to create comparable groups and calculates the *P*-value in a log-rank test, a *P*-value smaller than 0.05 is considered as statistically significant.

### Identification of MYC regulated super enhancer genes

To identify the super enhancer containing genes that are regulated by MYC in osteosarcoma. The MYC ChIP-seq and the control input data were downloaded from GSE77356.^15^ The fastq sequence reads were aligned to genome and then the MYC binding sites were identified by MACS2^28^ and annotated using PAVIS.^29^ The super enhancer regions in U2OS osteosarcoma cells were attained from the H3K27ac ChIP-Seq data (GSE87831)^16^ and annotated using PAVIS. Then the overlapping genes identified in both MYC ChIP-Seq and H3K27ac ChIP-Seq were defined as the MYC regulated super enhancer genes in osteosarcoma.

### Cell proliferation and apoptosis assays

The cell proliferation and apoptosis were determined as previously described.^30,31^ Briefly, to study the role of super enhancer inhibitors in osteosarcoma cell proliferation, U2OS, 143B, and BJ cells were treated with different concentrations of JQ1 (APExBIO A1910), THZ1 (APExBIO A8882), and DMSO and planted into 96-well cell culture plate at 1 000 cells/well, then cell proliferation was measured using CellTiter 96® AQueous One Solution Cell Proliferation Assay (Promega G3582) by OD490 at day 4 after treatment. For cell apoptosis assay, U2OS cells were treated with 500 nmol·L^-1^ JQ1, 100 nmol·L^-1^ THZ1 or DMSO for 48 h, then the cell apoptosis was measured by the Caspase 3/7 activities using the Caspase-Glo® 3/7 Assay Systems (Promega G8090) following the manufacturer’s instructions.

### Cell migration and invasion assays

The cell migration and invasion of U2OS osteosarcoma cells were performed as previously described.^32,33^ Briefly, wound healing assay was used to determine the roles of super enhancer inhibitors in regulation of osteosarcoma. U2OS cells were cultured in regular mediumto 100% confluence and then further cultured for another 24 h in low-serum (2%) medium. Then scratches were induced and cells were treated with 50 nmol·L^-1^ THZ1 and 250 nmol·L^-1^ JQ1 in low serum medium for 24 h. Cell images were then acquired to monitor the cell migration. To study the role of super enhancer inhibitors in osteosarcoma invasion, U2OS osteosarcoma cells were treated with 500 nmol·L^-1^ JQ1, 100 nmol·L^-1^ THZ1, or DMSO for 24 h, then 50 000 cells were seeded into the Corning™ BioCoat™ Matrigel™ Invasion Chamber (Corning 354480). The cell images and invasive cells were acquired and quantified after 16 h.

### Tumor xenograft model

Ten male athymic nude mice were maintained according to the protocols approved by the Shenzhen University Animal Care Commission. Animal experiments were performed in agreement with the guidelines of Animal Care Commission and the National Institutes of Health (NIH). For each mouse, five million U2OS cells were mixed with Matrigel (BD Biosciences) and subcutaneously injected in the dorsal flank of nude mice. Drug treatment was started one week after injection when the tumors reached an average volume of 100 mm.^3^ All mice were divided into two groups (*n* = 5 in each group), including vehicle control group (10% DMSO in D5W) and THZ1 treatment group (10 mg·kg^-1^ twice daily). Mice were treated with different conditions for 4 weeks and then euthanized for further analysis. The tumor volume (in mm^3^) was calculated using the formula: *V* = 1/2 (width^2^ × length).

### Immunohistochemistry and western blot

Immunohistochemistry was conducted as previously described.^34^ Ki-67 (Abcam, ab15580) antibody was diluted at the ratio of 1:200 and the slides were incubated with anti-Ki-67 antibody overnight at 4 °C and developed with streptavidin peroxidase conjugated approach. Western blot was performed as previously described.^31^ MYC (Cell Signaling Technology, 5605, 1:1 000) and β-Actin (Abcam, ab8229, 1:5 000) antibodies were used for the detection of MYC and β-Actin expression by Western blotting.

### High throughput RNA sequencing and data analysis

U2OS osteosarcoma cells were treated with 100 nmol·L^-1^ THZ1 or DMSO for 48 h, then total RNA samples were isolated using the Trizol according to the manufacturer’s instructions. Two microgram total RNA was used for the cDNA library construction using the TruSeq Stranded mRNA Library Prep Kit (Illumina RS-122-2101). Libraries were sequenced using Illumina NextSeq 500. The fastq sequence reads were aligned to genome using Tophat,^35^ then the differential gene expression between the THZ1 treated and control samples were analyzed using Cufflinks.^36^ Heat map was generated using HeatMap Builder (Stanford University). Student’s *t*-test was used for the statistical analysis.

## Electronic supplementary material

Supplementary Table 1
